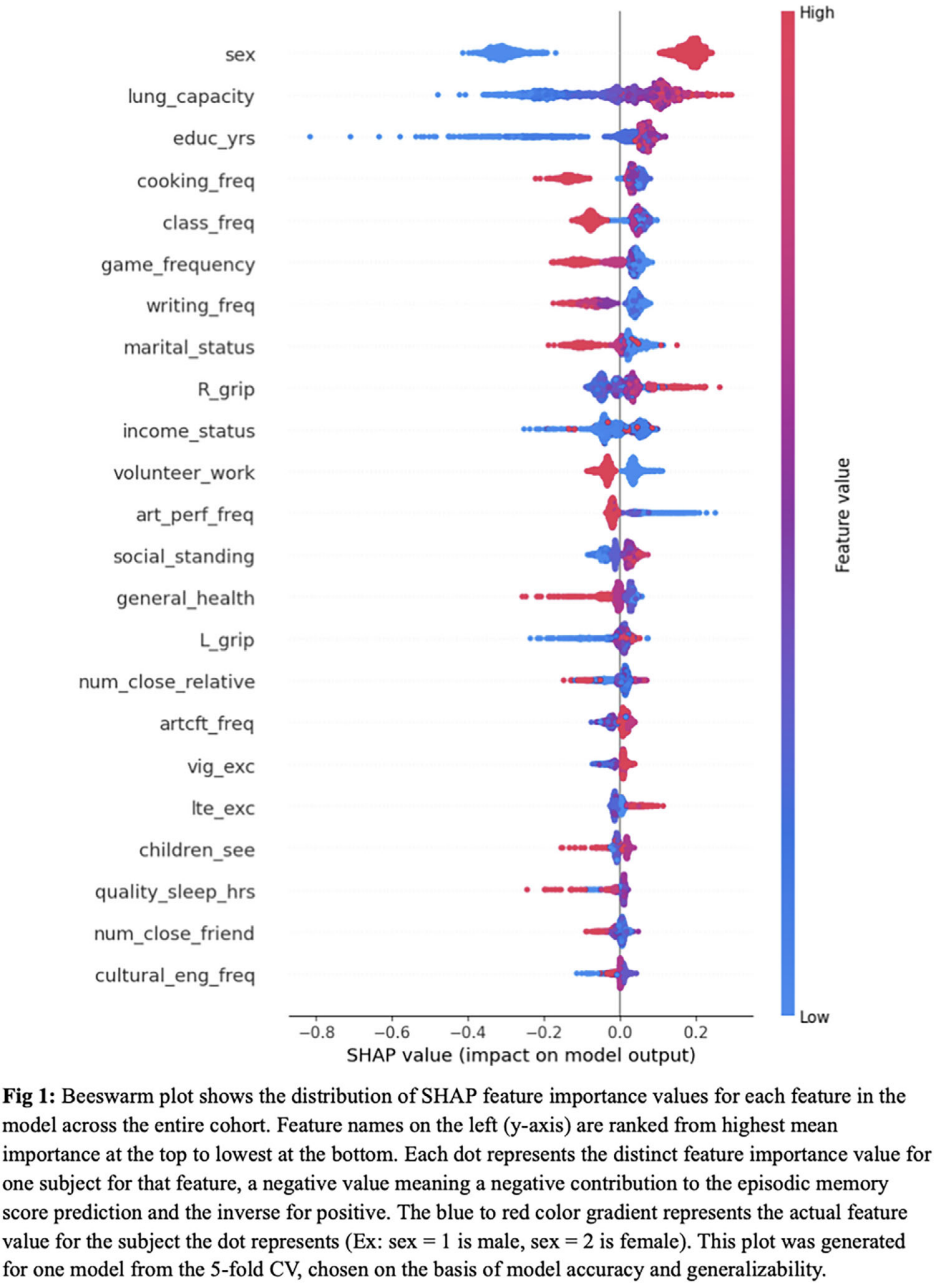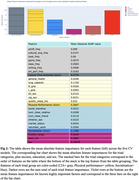# Lifestyle Factors’ Influence on Episodic Memory: A Gradient Boosted Tree Analysis

**DOI:** 10.1002/alz.095791

**Published:** 2025-01-09

**Authors:** Addison Berg, Stephanie Sinclair, Ashley Acosta‐Parra, Ana Nazmi Glosson, Cesar Moreno, Evan Fletcher

**Affiliations:** ^1^ UC Davis, Davis, CA USA

## Abstract

**Background:**

Comprehending the role of participatory lifestyle factors influencing the aging process and subsequent cognitive outcomes is imperative for postulating ways to combat cognitive decline. Recent research points to the *independent* impacts of leisure time activities (LTAs), physical performance (measures of physical fitness) and socialization (relationships and interaction frequency) on healthy cognitive aging, although the relative importance of this triad of factors is poorly delineated.

**Method:**

From baseline data of two studies examining cognitive aging in older, diverse populations— “Study of Healthy Aging in African Americans” and “Kaiser Healthy Aging and Diverse Life Experiences,” we scraped subjects’ self‐reported data and Spanish and English Neuropsychological Assessment Scales (SENAS) measured cognitive performance scores. Lifestyle survey questions revealed features that well‐represented each category in the triad. These features were used as model inputs, along with sex, education, and income. The cohort consisted of n = 1684 subjects, each characterized by 23 features, after removal for missingness. We applied a grid search algorithm with 5‐fold cross‐validation to tune the hyperparameters of a gradient boosted decision tree model. Optimized for accuracy and generalizability, these parameters defined the model to predict episodic memory scores. Per prediction, relative feature importances were computed as Shapley values. Mean absolute importances (global importance) for the full cohort were derived from the relative values. For triad comparison, summated global importance of the features for each lifestyle category was calculated.

**Result:**

Group importance ranking revealed aggregated LTAs with the largest contribution to episodic scores, followed by physical performance, then socialization. Notable LTAs included complex cooking, taking classes, and gaming. Although no individual mean absolute feature importance surpassed that of sex, the aggregate importance of the LTA and physical performance groups did; all categories exceeded education and income.

**Conclusion:**

Our major finding indicates that, beyond the typical factors of interest for episodic memory and healthy cognitive aging, the way one spends their free‐time has a strong association with these outcomes, and suggests LTAs are the most important. It’s worth noting that these three domains may overlap (ex. recreational sporting activities). This research may influence public health guidelines for promoting healthy cognitive aging.